# Poor Hematopoietic Stem Cell Mobilizers in Multiple Myeloma: a Single Institution Experience.

**DOI:** 10.4084/MJHID.2010.016

**Published:** 2010-06-21

**Authors:** Guillermo J. Ruiz-Delgado, Avril López-Otero, Ana Hernandez-Arizpe, Aura Ramirez-Medina, Guillermo J. Ruiz-Argüelles.

**Affiliations:** 1 Centro de Hematología y Medicina Interna de Puebla. Puebla, MEXICO; 2 Laboratorios Clínicos de Puebla. Puebla, MEXICO; 3 Universidad Popular Autónoma del Estado de Puebla

## Abstract

In a single institution, in a group of 28 myeloma patients deemed eligible for autologous transplant, stem cell mobilization was attempted using filgrastim: 26 individuals were given 31 autografts employing 1–4 (median three) apheresis sessions, to obtain a target stem cell dose of 1 x 10^6^ CD34 +ve viable cells / Kg of the recipient. The median number of grafted CD34 cells was 7.56 x 10^6^ / Kg of the recipient; the range being 0.92 to 14.8. By defining as poor mobilizers individuals in which a cell collection of < 1 x 10^6^ CD34 viable cells / Kg was obtained, a subset of eight poor mobilizers was identified; in two patients the autograft was aborted because of an extremely poor CD34 +ve cell yield (< 0.2 x 10^6^ CD34 +ve viable cells / Kg of the recipient) after four apheresis sessions. The long-term overall survival of the patients grafted with > 1 x 10^6^ CD34 +ve viable cells / Kg was better (80% at 80 months) than those grafted with < 1 x 10^6^ CD34 +ve viable cells / Kg (67% at 76 months). Methods to improve stem cell mobilization are needed and may result in obtaining better results when autografting multiple myeloma patients.

## Introduction:

High-dose chemotherapy followed by autologous stem cell transplantation (SCT) is considered standard of care for patients with multiple myeloma (MM).^1^,^2^ In this setting, autologous peripheral blood stem cells (PBSC) have largely replaced autologous bone marrow as the source of stem cells. Advantages of autologous PBSC grafts over bone marrow grafts include faster engrafment after high-dose chemotherapy, reduced contamination with tumor cells^3^ and lower morbidity and mortality.^3^–^5^

Hematopoietic stem cell mobilization is generally accomplished by administration of hematopoietic growth factors like granulocyte colony stimulating factor (G-CSF)^1^–^5^ or a combination of myelosuppressive chemotherapy and hematopoietic growth factors. A number of factors have been reported to impact progenitor cell mobilization but the predictive factors vary from one study to the other.

The purpose of this study is to analyze the prevalence of poor mobilization in individuals with MM, prospectively studied and grafted in a single institution using the same autografting procedure.

## Patients and Methods:

### a) Patients:

Patients with MM diagnosed and autografted at the Centro de Hematología y Medicina Interna de Puebla, a part of the Clínica RUIZ, in Puebla, México between August 1993 to September 2008 were prospectively included in the study. The diagnosis of MM was based on the following findings:^1^–^2^,^4^ a) Increased numbers (more than 30%) of abnormal, atypical or immature plasma cells in the bone marrow or histologic proof of plasmacytoma; b) presence of an M-protein in the serum or urine; or c) Bone lesions consistent with those of multiple myeloma. Individuals with plasma cell reactions to connective tissue disorders, liver disease, metastatic carcinoma or chronic infections were not included, nor patients with monoclonal gammopathy of undetermined significance,^3^ smoldering multiple myeloma, solitary plasmacytoma or plasma cell leukemia. Individuals with primary amyloidosis (AL) were included only if features of MM predominated.^6^,^7^ All patients were autografted in the *Centro de Hematología y Medicina Interna de Puebla, México,* and had received a median of 4 (range 1–7) prior chemotherapy regimens (**Table 1**). Normal renal and liver function tests were needed for inclusion as well as informed consent approved before any procedure.

### b) PBSC mobilization and apheresis:

The PBSC mobilization schedule was started at least 30 days after the last dose of chemotherapy. Subcutaneous G-CSF (10 ug / Kg / day / 5 days) was given for mobilization of stem cells, starting day - 7. Using either a peripheral vein or a Majurkar-type subclavian catheter, the apheresis procedures were performed on days – 4 – 3, – 2 and – 1, using a Haemonetics V-50 PLUS machine (*Haemonetics* Corporation, Braintree MA) or a *Baxter* C-3000 PLUS machine (*Baxter* Healthcare, Deerfield IL), and the Spin-Nebraska protocol.^8^ The endpoint of collection was the processing of 5000 – 7000 ml of blood / m^2^ in each of the apheresis procedures, in order to obtain at least 3 x 10^8^ mononuclear cells and / or 1 x 10^6^ viable CD34 cells / Kg of the recipient’s weight.^1^,^3^–^5^

### c) Conditioning and autografting:

Intravenous melphalan, 200 mg/m^2^ in a single I.V. dose was used on day – 1 in all patients. Ondansetron (8 mg i.v. every 12 h after chemotherapy), ciprofloxacin (250 mg. *bid*) and fluconazole (200 mg *bid*) were used in all patients. G-CSF (10 ug / Kg / day / 5 days) was re-started on day +5 and used until granulocytes were greater than 0.5 x 10^9^/L. Antibiotics and antimycotics were used until granulocytes were above 0.5 x 10^9^/L. All patients had daily laboratory workup and clinical studies.

### d) Apheresis product preservation, studies and infusion:

The products of the apheresis and 1 ml aliquots were kept in ACD-A (Baxter Healthcare, Deerfield IL) at 4°C, in 300 ml transfer packs (Baxter Healthcare, Deerfield IL) composed of gas impermeable, polyvinyl chloride plastic film for up to 96 hours. Enumeration of the total white mononuclear cells (MNC) and CD34 +ve cells was done by flow-cytometry^9^ in an EPICS Elite ESP apparatus (Coulter Electronics, Hialeah, FL), using for the latter subpopulation the anti-CD34 monoclonal antibody HPCA-2 (Becton Dickinson, San José CA), gating in propidium iodide-excluding CD45(+) MNC population according to forward and 90° angle light scattering. Additional viability studies of the MNC prior to its re-infusion used propidium iodide exclusion and anti-cell antibodies on a flow cytometer. No purging procedures were performed. The apheresis products obtained on days – 4, – 3, – 2 and – 1 were reinfused to the patients on days 0, +1, +2 and +3 respectively after keeping them in the conventional blood bank refrigerator at 4 degress centigrade.^3^,^4^

### e) Criteria for assesment of the mobilization procedures:

One to four apheresis sessions were performed to obtain a target stem cell dose of 1 x 10^6^ CD34 +ve viable cells / Kg of the recipient. Cases in which less than this amount of CD34 +ve progenitor cells were obtained after four apheresis sessions were classified as “poor” mobilizers.

### f) Response assessment:

Very good partial response (VGPR) was defined as a decrease of 90% in the serum paraprotein level. Partial remission (PR) as a decrease of 50% in the serum paraprotein level, and minimal response (MR) as a decrease of 25% in the serum paraprotein level.^1^

## Results:

### a) Patients:

All patients with multiple myeloma less than 70 years are offered an autograft in our institution; 28 consecutive patients were deemed eligible for autologous transplant and attempted stem cell mobilization; there were 13 females and 15 males. Since our autografting procedure does not involve hematopoietic stem cell freezing,^1^,^4^–^5^ the procedure was aborted if less than 0.5 x 10^6^ CD34 +ve viable cells / Kg of the recipient were collected; accordingly, the autograft was aborted in two patients because of a very poor CD34 +ve cell yield (less than 0.2 x 10^6^ CD34 +ve viable cells / Kg of the recipient) after four apheresis sessions; one patient had developed an acute myelogenous leukemia after being treated with oral melphalan during 51 months and failed to mobilize after entering a complete remission of the leukemia; he was later on given an allogeneic graft with umbilical cord cells and remains disease-free 75 months after attempting the autograft.^10^ The other patient had received an autologous graft using intravenous melphalan 51 months before attempting the second autograft; after failing mobilization with G-CSF he was later on mobilized with G-CSF plus plerixafor and subsequently re-autografted succesfully.^11^

In the group of 26 patients who were autografted, the median age was 54 years (range 42 to 66). The type of paraprotein was IgG in 16 cases, IgA in 6 cases, light chain disease in 2 cases (both kappa). According to the *International Staging System* (ISS),^12^ 21 patients were in stage I, 3 in stage II and 2 in stage III. All patients had been treated before the autograft: Six with thalidomide / dexametasone (thal/dex), 11 with bortezomib-containing regimens, 9 with vincristine / adriamycin / dexamethasone (VAD) and 5 with other schedules. No patient had received radiation therapy as part of the previous treatment and in all cases the autologous graft was performed as part of the initial therapy.

### b) PBSC mobilization and apheresis:

A median of three apheresis sessions were needed to collect a minimum of 1 x 10^6^ CD34 +ve viable cells / Kg of the recipient;^1^ the range was 2 to 4 sessions to obtain enough CD34 +ve cells. Circulating CD34 +ve cells were not enumerated prior to the apheresis procedures.

### c) Conditioning and autografting:

All patients were conditioned with a single dose of intravenous melphalan, 200 mg/ m^2^. In cases in which less than 1 x 10^6^ CD34 +ve viable cells / Kg of the recipient, defined as poor mobilizers, the dose of melphalan was adjusted to 180 mg/m^2^ (90% of the planned dose).

### d) Apheresis product studies:

The median number of transplanted CD34 +ve viable cell was 7.56 x 10^6^ CD34 +ve viable cells / Kg of the recipient; the range was 0.92 to 14.8. In all cases the viability of the CD34 cells was above 85% prior to being reinfused to the patients.

### e) Assesment of the mobilization procedures:

One to four (median three) apheresis sessions were performed to obtain a target stem cell dose of 1 x 10^6^ CD34 +ve viable cells / Kg of the recipient. In six cases, less than 1 x 10^6^ CD34 +ve viable cells / Kg of the recipient were obtained, in 16 cases 1 to 2 x 10^6^ CD34 +ve viable cells / Kg of the recipient, whereas in 9 cases more than 2 x 10^6^ CD34 +ve viable cells / Kg of the recipient.

### f) Engraftment and response:

In the whole group of patients, the time to achieve more than 0.5 x 10^9^/L granulocytes had a median of 27 days (range 0 to 53), whereas the time to recover more than 20 x 10^9^/L platelets had a median of 37 days (range 0 to 73). Of the 26 patients autografted, 19 achieved a complete remission, 6 a very good partial remission, 5 a partial remission and one a minor response. The 100-day mortality was 9.6% (3 of 31 transplants); Two patients died at day +9 and +11 as a result of sepsis during granulocytopenia and one died at day +100 due to a myocardial infarction; they had received 2.5, 1.5 and 3.4 x 10^6^ CD34 +ve viable cells / Kg, respectively. The overall median post-transplant survival (OS) has not been reached, being above 80 months, whereas the 80-month OS is 77%. Relapses presented in 11/31 autografts, a median of 34 months after the procedure; five patients were rescued with a second autograft and two were given an allogeneic grafts. The autograft procedure was started in an outpatient basis in all instances, however four patients where admitted to the hospital as a result of: amebic colitis, *Pseudomona aeruginosa* sepsis, cerebrovascular episode and soft-tissue abscess, one case each.

**Figure 1.**
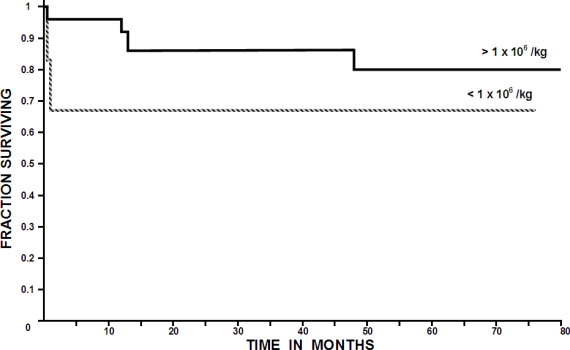
Overall survival of the 26 multiple myeloma patients given 31 autografts, and divided according to the number of hematopoiteic stem cells autografted (more or less than 1 x 10^6^ CD34 +ve viable cells / Kg of the recipient). The differences are not statistically significant.

In the whole group of 31 autografts, a subset of six poor mobilizers was identified. The **table 1** shows the salient data of these six poor mobilizers compared with those of the 25 autografts in which a better mobilization was obtained. It is interesting that the long-term overall survival of the multiple myeloma patients allografted with more than 1 x 10^6^ CD34 +ve viable cells / Kg was better (80% at 80 months) than those autografted with less than 1 x 10^6^ CD34 +ve viable cells / Kg (67% at 76 months); the differences however are not statistically significant according to the log-rank chi square method most likely as a result of the low number of patients included in the study, see **table 1** and **figure 1**. There were not significant differences in time to recover granulocytes and platelets between patients receiving more or less than 1 x 10^6^ CD34 +ve viable cells / Kg.

## Discussion:

A number of factors have been reported to impact stem cell mobilization. These include patient age, weight, diagnosis, type and number of prior chemotherapeutic regimens administered, bone marrow involvement with the malignancy at baseline, extent of cell recovery from previous chemotherapy at the time of starting mobilization treatment, prior radiation therapy, time from diagnosis to harvest, and disease status.^13^–^15^ Other factors include type of cytokine used and type and dose of chemotherapy regimen, if any, for mobilization.^13^–^15^

The number of CD34 +ve hematopoietic stem cells required to successfully conduct an autograft has not been clearly defined. Some authors have described that a minimum of 2 x 10^6^ CD34 +ve viable cells / Kg of the recipient is required to rescue the hematopoiesis of the patient.^13^ We have shown that a minimum of 1 x 10^6^ CD34 +ve viable cells / Kg of the recipient may be useful to successfully autograft patients with multiple myeloma^1^ or other diseases.^3^–^4^ This apparent discrepancy may stem from several factors, one of them being the enumeration methods: we have also shown previously that a considerable variation might be due to the selection of antibodies, staining protocols, and acquisition strategies for the flow cytometric enumeration of these cells which may account even for two-fold differences;^16^ both inter-instrument and inter-protocol variation provide explanation for the redundantly reported discrepancies concerning the numbers of CD34 cells that suffice to secure hematopoietic grafting and to define poor mobilizers.^16^ Accordingly, the exact incidence of poor mobilizers in the setting of autologous grafting is unknown and has varied in the literature between 5 to 40% in different subsets of patients.^13^ Using a lower threshold, we have found a prevalence of 24% (8 of 33 autografts) of poor mobilizers, which only in two cases led into aborting the autografting procedure. With the limitations of a small study, we found that certain features of the patients and/or disease were associated with a defective mobilization: Previous treatment with oral melphalan, previous autograft with intravenous melphalan and previous treatment with bortezomib. The low number of patients included in each subset of treatments makes hard to know of prior exposure to certain drugs *per se* interfered with mobilization, or whether patients exposed to some agents had other features that would compromise mobilization. On the other hand, it should be mentioned that some patients who had been treated previously with oral melphalan could be successfully autografted; accordingly, we believe that attempts of autografting should be tried even in patients previously treated with oral melphalan.

In order to improve the mobilization techniques for stem cell autografting, several approaches have been tried. The mobilization seems to be better if both chemotherapy and growth factors are employed.^17^ Recently, Dugan *et al.* showed that plerixafor can safely be added to chemotherapy-based mobilization regimens and may accelerate the rate of increase in CD34 +ve cells on the second day of apheresis^18^ whereas Di Persio *et al.* have shown similar results in patients with multiple myeloma.^19^ Further studies are warranted to evaluate the effect of this drug in combination with chemomobilization on stem cell mobilization and collection on the first and subsequent days of apheresis, and its impact on resource utilization and results improvement.^19^,^20^ Since there are data suggesting that failure of mobilization in myeloma patients results in increased expenses and increased use of resources,^21^ methods to predict the failure to mobilize have been suggested.^22^ Studies to analyze the balance of the costs of improved mobilization methods and the costs of failing to mobilize patients with myeloma who are to be autografted are mandatory to further explore this field, specially in developing countries, where autografting patients with multiple myeloma is still a cheaper therapeutic option than the use of the novel anty-myeloma drugs.^23^

## Conclusions:

In summary, we have found that in patients with multiple myeloma, using a threshold of 1 x 10^6^ CD34 +ve viable cells / Kg of the recipient has allowed us to define a suboptimal mobilization which presents in one quarter of patients and seems to be associated with previous treatment with oral melphalan, previous autograft with intravenous melphalan and previous treatment with bortezomib, and that the long term overall survival of the poor mobilizers seems to be worse than that of individuals grafted with 1 x 10^6^ CD34 +ve viable cells / Kg of the recipient. Efforts directed to improve stem cell mobilization seem to be warranted and could result in improving the results of autografting and the prognosis of patients with multiple myeloma.^24^

## Figures and Tables

**Table 1:** Salient features of the 31 autografts divided into two groups according to the number of hematopoietic stem cells autografted.

	**n**			
**Transplants**	**31**			

**Patients**	**26**			

	CD34 +ve MNC grafted			
	< 1 x 10^6^ /kg		> 1 x 10^6^ /kg	

Transplants	6		25	

Age	Median 54	Range 43 – 65	Median 55	Range 44 – 66

Paraprotein				
IgG	5		15	
IgA	1		6	
Light chaín only	0		2	
Non secretory	0		2	

Apheresis sessions	Median 3	Range 3 – 4	Median 3	Range 2 – 4

Previous treatment:				
Thal / Dex	1 (16%)		6 (24%)	
VAD	0		10	
Bortezomib	4 (66%)		6 (24%)	
Oral melphalan	2 (33%)		3 (12%)	
Autograft	2 (33%)		2 (8%)	
Others	1		3	
Overall survival	67% at 76 mos.		80% at 80 mos.	

MNC = mononuclear cells; Thal = thalidomide, Dex = dexametasone; VAD = vincristine, adriamycyn and dexametasone; mos = months.
